# COVID-19 vaccine attitudes among mental health professionals in the WHO’s global clinical practice network

**DOI:** 10.1371/journal.pmen.0000018

**Published:** 2024-06-26

**Authors:** Cary S. Kogan, Dan J. Stein, José A. Garcia-Pacheco, Tahilia J. Rebello, Madeline I. Montoya, Rebeca Robles, Brigitte Khoury, Maya Kulygina, Chihiro Matsumoto, Jingjing Huang, María Elena Medina-Mora, Oye Gureje, Pratap Sharan, Wolfgang Gaebel, Shigenobu Kanba, Howard F. Andrews, Michael C. Roberts, Kathleen M. Pike, Min Zhao, José Luis Ayuso-Mateos, Karolina Sadowska, Karen Maré, Keith Denny, T. Scott Stroup, Geoffrey M. Reed

**Affiliations:** 1 School of Psychology, University of Ottawa, Ottawa, Ontario, Canada; 2 Department of Psychiatry & Neuroscience Institute, SAMRC Research Unit on Risk & Resilience in Mental Disorders, University of Cape Town, Cape Town, South Africa; 3 Centro de Investigación y Docencia Económica, Mexico City, Mexico; 4 Columbia University Vagelos College of Physicians and Surgeons, New York, New York, United States of America; 5 National Institute of Psychiatry Ramón de la Fuente Muñiz, Center for Global Mental Health Research, Mexico City, Mexico; 6 Department of Psychiatry, American University of Beirut Medical Center, Beirut, Lebanon; 7 Training and Research Centre, Mental-Health Clinic No.1 Named After N.A. Alekseev, Moscow, Russian Federation; 8 Japanese Society of Psychiatry and Neurology, Tokyo, Japan; 9 Shanghai Mental Health Center and Shanghai Jiao Tong University School of Medicine, Shanghai, People’s Republic of China; 10 National Institute of Psychiatry Ramón de la Fuente Muñiz, Center for Global Mental Health Research, Mexico City, Mexico; 11 Faculty of Psychology, National Autonomous University of Mexico, Mexico City, Mexico; 12 Department of Psychiatry, WHO Collaborating Centre for Research and Training in Mental Health, Neuroscience, Drug and Alcohol Abuse, University of Ibadan, Ibadan, Nigeria; 13 Department of Psychiatry, All India Institute of Medical Sciences, New Delhi, India; 14 Medical Faculty, Department of Psychiatry, WHO Collaborating Centre DEU-131, LVR-Klinikum Düsseldorf, Heinrich-Heine University, Düsseldorf, Germany; 15 Kyushu University, Fukuoka and Japan Depression Center, Tokyo, Japan; 16 Departments of Biostatistics and Psychiatry, Columbia University and New York State Psychiatric Institute, Columbia University Medical Center, New York, New York, United States of America; 17 Clinical Child Psychology Program, University of Kansas, Lawrence, Kansas, United States of America; 18 Departments of Psychiatry and Epidemiology, Columbia University Irving Medical Center, New York, New York, United States of America; 19 Department of Psychiatry, Instituto de Investigacíon Sanitaria La Princesa, Instituto de Salud Carlos III, Universidad Autónoma de Madrid, Centro de Investigacíon Biomédica en Red de Salud Mental (CIBERSAM), Madrid, Spain; 20 Department of Sociology and Anthropology, Carleton University, Ottawa, Ontario, Canada; 21 Department of Psychiatry, Columbia University Vagelos College of Physicians and Surgeons, New York, New York, United States of America; Hamdard University - Islamabad Campus, PAKISTAN

## Abstract

Although COVID-19 vaccines have demonstrated efficacy, there is variability in health professionals’ attitudes towards these agents. Factors associated with mental health professionals’ attitudes towards COVID-19 vaccination are not well understood. We investigated these factors by administering a newly developed measure, the COVID-19 Vaccine Attitudes Questionnaire (C-VAQ), to members of the World Health Organization’s Global Clinical Practice Network (GCPN) of mental health professionals. 1,931 GCPN members representing all world regions participated between July 28 and September 7, 2021. Mental health professionals’ attitudes towards COVID-19 vaccination were assessed in one of five languages (Chinese, English, French, Japanese, Russian, or Spanish) using the C-VAQ. Internal consistency, factor structure, and predictive validity of the C-VAQ were examined, and a multiple-linear regression model was employed to assess C-VAQ score predictors, including sociodemographic variables (age, gender, WHO region, country income level, profession, and years of professional experience) as well as country mortality rate and the stringency of each country’s response to COVID-19. The C-VAQ demonstrated good internal consistency and external validity. Items loaded on to a single factor. Having received a COVID-19 vaccine, higher country mortality rate, and higher stringency index was significantly associated with more positive vaccine attitudes. Lower age, residing in a low-and-middle income country, and living in Asia were all was significantly associated with less positive vaccine attitudes. The C-VAQ scores were negatively correlated with the number of concerns about the COVID-19 vaccination. The C-VAQ was useful in demonstrating the extent to which additional work is needed to improve mental health professionals’ attitudes towards COVID-19 vaccines globally. Relatively poorer attitudes toward vaccination among some mental health clinicians around the world suggests the need for broad, multi-pronged interventions.

## Introduction

Despite the paramount importance of vaccines for improving global health, vaccine hesitancy has a long history, and persists worldwide [1]. Given the deleterious effects of the COVID-19 pandemic to individuals and society, and the positive impact of COVID-19 vaccination, it is particularly crucial to understand those factors that affected COVID-19 vaccine acceptance and hesitancy among different groups including mental health professionals. A recent systematic review of 51 studies found that vaccine hesitancy and avoidance were highly prevalent, not only in the general public, but also among health care workers, emphasizing the need to further investigate obstacles to vaccine uptake, and to develop appropriate evidence-based interventions [2]. Attitudes are causally linked to health intentions and behaviour with a medium effect size [3], emphasizing the importance of measuring these cognitions and considering them for targets of interventions intended to increase vaccination rates. Therefore, the availability of a self-report measure of COVID-19 vaccine knowledge and attitudes could be useful in achieving this important public health goal.

COVID-19 negatively impacts physical and mental health, and given the important role of health care workers, including mental health professionals, in providing interventions and in vaccine advocacy, it is particularly important to understand vaccine attitudes in this group. A recent scoping review of vaccine intentions in health care workers identified 26 papers and emphasized a range of concerns about vaccine safety and efficacy [4]. Our current understanding of predictors of vaccine hesitancy in health care workers is limited, with even fewer data available about vaccine hesitancy in mental health professionals [4]. The importance of attitudes toward vaccines in this group is underscored by evidence that individuals with serious mental illness may be at heightened risk of infection and increased mortality [5]. Furthermore, protection of an already fragile and understaffed mental health workforce is a priority [6].

The World Health Organization’s Global Practice Network (GCPN) is comprised of more than 16000 mental health professionals representing a diverse range of geographic regions and settings, and provides a unique opportunity to investigate professional’s knowledge and attitudes from a global perspective. We aimed to investigate these professionals’ knowledge about and attitudes toward vaccination by administering a new 8-item measure, the COVID-19 Vaccine Attitudes Questionnaire (C-VAQ), to members of the GCPN. We investigated whether sociodemographic variables, vaccination status, as well as country mortality rate and stringency index at the time of data collection predicted C-VAQ scores.

## Material & methods

The WHO’s Global Clinical Practice Network (GCPN) and the “Longitudinal COVID-19 Survey of Mental Health Professionals Project (LoCS MHP)” have been described elsewhere in more detail [7]. Briefly, the GCPN is a multilingual network of mental health clinicians, mainly psychiatrists and psychologists, representing all regions of the world [8]. To be eligible for the GCPN, members must have completed their professional training and be formally authorized to provide mental health services in their countries. At the time of the study in July 2021, the GCPN comprised more than 16000 participants from 163 countries, with excellent representation of clinicians working in low and middle-income countries. The “LoCS MHP” is an international collaboration funded by the Canadian Institute of Health Research. The “LoCS MHP” is a three-wave longitudinal internet-based survey study on the effects of the COVID-19 pandemic on numerous facets of mental health professionals’ practice and well-being (e.g., [9]). Questions related to vaccine attitudes and concerns were included in the third wave of data collection, which occurred between July 28 to September 7, 2021. After the initial invitations to participate were sent out, follow up email reminders were sent to those GCPN members who had not yet responded one week after the initial invitation and one week after the first reminder. The survey was disseminated on line using Qualtrics (Provo, UT, USA) and conducted in Chinese, English, French, Japanese, Russian, and Spanish. Survey questions were developed in English and translated by experts fluent in these languages and affiliated with the GCPN’s International Advisory Group.

GCPN members who at the time of registration had indicated that they were proficient in the corresponding study language were sent an email invitation containing an individualized survey link. Participants had previously attested to their language proficiency as part of the information provided when they registered as a member of the GCPN. Upon accessing the link, participants were asked to read a description of the study and provide their consent to participate.

Several sources of bias are inherent in questionnaire research including sampling bias, response bias, and social desirability [10]. GCPN members are from all regions of the world but may not be a representative sample. In order to ensure that the sample of GCPN members who responded to the survey were representative of the overall membership, differences in demographic variables were examined across non-respondents and respondents. To avoid the potential of response bias, the C-VAQ includes four reverse scored items. Internal consistency as well as the mean, range, and standard deviation of scores were examined to ensure that responses were non-random. Social desirability to conform with the majority view in the medical community that vaccination againt COVID-19 is beneficial, participants may have presented themselves in a more vaccine-positive light than reflects their actual beliefs. To control for such a bias, vaccination status was included as a variable in the analyses.

### Ethics statement

The study was approved by the Institutional Review Board at Columbia University/New York State Psychiatric Institute (Registration number: #6886) and the University of Ottawa (Registration number: H-06-20-5973). Participants, who were all practicing clinicians, provided their consent electronically in the Qualtrics survey by selecting “Yes” after being provided the opportunity to review the approved consent form.

### COVID-19 Vaccine Attitudes Questionnaire (C-VAQ)

#### Questionnaire development

The COVID-19 Vaccine Attitudes Questionnaire (C-VAQ) was developed through a review of the existing literature at the time including media reports and gray literature as well as group discussions and an expert review by the LoCS MHP International Advisory Group (IAG). Item selection and inclusion was based on the common causal factors of theories of health behaviour change [3], which posit that attitudes, self-eficacy, and norms each contribute to intention to change and actual change in health behaviour. The IAG for this project comprised internationally known psychiatrists and psychologists representing all WHO global regions and many of the countries hardest hit by the pandemic (Canada: CSK, KD, P.R. China (MZ), Germany (WG), India (PS), Japan (SK), Mexico (MEM), Nigeria (OG), Russian Federation (MK), Spain (JA), and USA (KP, GMR).

Group discussions with the IAG took place virtually on three occasions and were guided by those themes identified by two of the co-authors (GMR and TJR) from their literature search. The C-VAQ addresses themes that appear in the emergency literature on COVID-19 vaccine knowledge and uptake. Themes included: 1) confidence in safety and efficacy of the vaccine, 2) beliefs about the vaccine, 3) knowledge about the vaccine, and 3) feelings about the vaccine. The IAG were tasked with creating a measure that could easily be administed. They discussed and reached consensus on 8 items (see Table 1). The C-VAQ was then reviewed by expert members of the IAG who determined that the 8 items selected met the requirements of content and face validity.

**Table 1:** Items of the COVID-19 Vaccine Attitudes Questionnaire (C-VAQ).

1. I am confident that the vaccine will protect me from COVID-19
2. I feel fortunate in having access to the vaccine
3. I trust the safety and effectiveness of the COVID-19 vaccine
4. The benefits of the vaccine are greater than the risks
5. The COVID-19 vaccine is not necessary for most people because most cases are mild or asymptomatic
6. Developing natural immunity after being exposed to COVID-19 is more effective than being vaccinated
7. It is not necessary to be vaccinated if you have already had COVID-19
8. There is not enough information about long term effects of the vaccine

The C-VAQ was administrated to participants. The C-VAQ comprised 8 items that were self-rated on a 5-point Likert-like scale. The C-VAQ has a total score range of 0 to 40, with the last four items reverse scored such that higher scores reflect greater confidence in the COVID-19 vaccine and vaccination process. These items assess knowledge of and attitudes towards COVID-19 vaccines. Confirmation of the content validity of the C-VAQ is suggested by the partial overlap of items with a longer, 39-item questionnaire, that was developed and validated for use in the general population using a mixed methods study design in India [11].

In addition to the C-VAQ, participants were asked a single question with twelve potential options from which they could select multiple responses to solicit their concerns related to the COVID-19 vaccine or the vaccination process (Table 2).

**Table 2:** COVID-19 concern question.

What concerns have you had about the COVID-19 vaccine? (Select all that apply)
1. I have had a bad reaction to vaccines in the past
2. I do not feel like I have enough information about the vaccine
3. I have received conflicting information about the vaccine
4. Concerns about contents of the vaccine
5. Concerns about short-term side effects of the vaccine
6. Concerns about severe adverse events due to the vaccine (e.g., blood clots)
7. Concerns about long-term side effects of the vaccine
8. Concerned that I might get COVID-19 through the vaccine
9. I do not trust pharmaceutical companies that have a financial interest in the vaccine
10. I do not believe the safety of a vaccine authorized on an emergency basis can be guaranteed
11. I do not trust my government to ensure that vaccines are safe
12. I do not trust the health system

### Predictor variables

Using a social ecological framework, factors associated with COVID-19 vaccine attitudes can be grouped into intrapersonal, interpersonal, institutional, community, and public policy factors [12]. In our survey, sociodemographic variables included age, gender, global region as defined by WHO, country income level, profession, and years of professional experience (Table 3). We also assessed whether people had been infected with COVID-19, and whether people had been vaccinated against COVID-19 with at least one dose. We summarized the number of concerns of the participants as a proxy for the degree of vaccine-related worries. Finally, we assessed organizational influences based on whether participants considered that the organization’s leadership communicated effectively to staff about vaccine availability and procedures, and made educational materials available about vaccine safety, efficacy, and side effects.

**Table 3:** Sociodemographic characteristics of participants (N = 1,931).

**Gender**	
Male	974 (50.4%)
Female	957 (49.6%)
**Language**	
English	870 (45.1%)
Spanish	369 (19.1%)
Japanese	237 (12.3%)
Russian	194 (10%)
French	164 (8.5%)
Chinese	97 (5.0%)
**WHO Region**	
African	81 (4.2%)
Americas-South	270 (14.0%)
Americas-North	237 (12.3%)
Eastern Mediterranean	39 (2.0%)
Europe	772 (40.0%)
South-East Asian	124 (6.4%)
Western Pacific-Asia	361 (18.7%)
Western Pacific-Oceania	47 (2.4%)
**Country**	
Japan	238 (12.3%)
Russian Federation	160 (8.3%)
United States of America	138 (7.1%)
Spain	126 (6.5%)
Other	1269 (65.7%)
**Income level**	
Low	16 (0.8%)
Lower-middle	211 (10.9%)
Upper-middle	585 (30.3%)
High	1119 (57.9%)
**Profession**	
Medicine	936 (48.5%)
Psychology	734 (38.0%)
Nursing	39 (2.0%)
Social Work	47 (2.4%)
Other	54 (2.8%)
Sex Therapy	3 (0.2%)
Speech Therapy	1 (0.1%)
Occupational Therapy	57 (3.0%)
Certified Peer Support Worker	1 (0.1%)
**Age**	
Mean (SD)	50.7 (11.6)
Median [Min, Max]	50 [25–89]
**Years of experience**	
Mean (SD)	20.3 (10.6)
Median [Min, Max]	20.0 [0–58]
**COVID-19 Vaccination Status**	
At least one vaccine dose	92.6%
Unvaccinated	7.1%

We calculated the average new COVID-19 mortality rate by country in which GCPN members were working at the time of data collection [13]. The stringency index, which is a composite measure based on nine indicators of the strictness of each country’s response to COVID-19 (e.g., stay-at-home orders, school and workplace closures, travel bans), was also included in the analyses. Stringency was rescaled to a value from 0 to 100, where 100 was set as representative of the strictest COVID-19 public health measures to control the spread of the virus [14].

### Statistical analyses

To avoid loss of data, we implemented a multiple imputation process for missing predictive variables using Bootstrap and Predictive Mean Matching [15]. In particular, data were imputed for the indices of total number of COVID-19 vaccination doses administered per 100 people in the total population [16], rate of new deaths per COVID-19 [17], and the stringency index [14].

The internal consistency (Cronbach’s alpha) and factor structure (polychoric correlation and Scree plot) of the C-VAQ were calculated. To assess predictors of C-VAQ scores, a multiple-linear regression model was employed, including sociodemographic variables (age, gender, WHO region, country income level, profession), COVID-19 variables (infection status, vaccination status, number of concerns), organizational variables (communication, education), mortality rate, and stringency index. All statistical calculations were performed with R statistical software version 3.6.1 using R studio version 2021.09.1+372.

## Results

### Sample characteristics

A total of 14,361 GCPN members were invited to participate in the study. A total of 2,012 people participated in wave 3 of the study, with 1,931 responding to those items related to vaccine acceptance, hesitancy and concerns. Therefore, a total of 13.4% of the GCPN members responded to and completed the study, a response rate that is consistent with previous studies conducted with the GCPN. The demographic and professional characteristics of these participants were tabulated (Table 3); they represent all WHO global regions, including 113 countries, and are similar to those of GCPN members as a whole. We analyzed whether there were differences between GCPN members who responded to the survey and those who did not. Responders and non-responders were not statistically significantly different on gender (p = .062) but were found to differ on age (p < .001, responders were on average 1.6 years older), years of experience (p < .001, responders had on average 1.3 more years of experience), profession (p < .001, more psychologists, 38% versus 30%, and fewer physicians 48.5% versus 53.6% were responders), WHO region (p < .001, fewer people from Asia responded, 18.3% compared to 23%) and income group (p < .001, fewer people from middle-income countries responded, 22% compared to 28%) (Table 1). The effect sizes for significant differences were in the very small range (0.05–0.13).

### Internal consistency and factor structure of the Vaccine Attitudes Questionnaire (C-VAQ)

**Fig 1:**
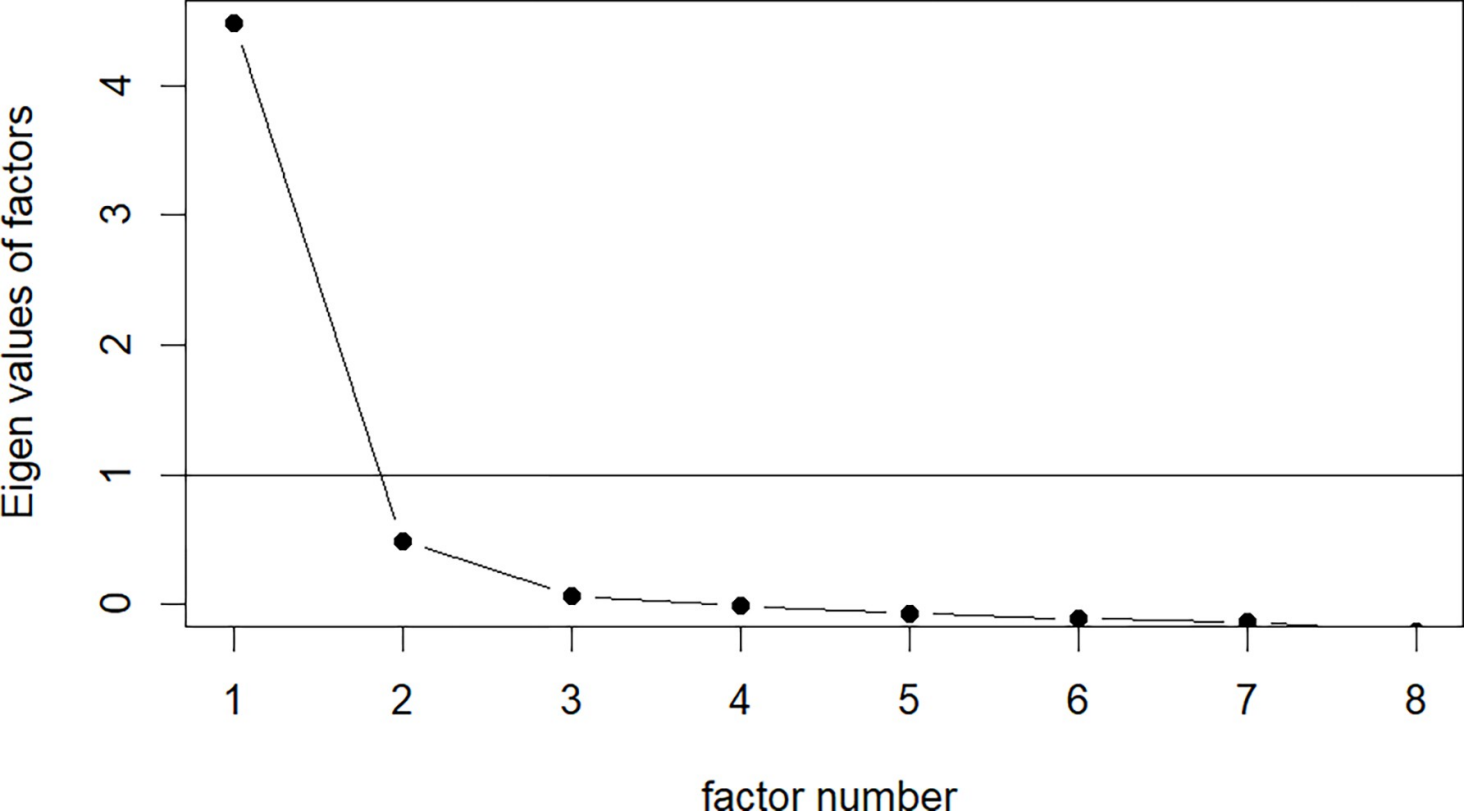
Scree plot for factors of the COVID- 19 Attitudes Questionnaire (C-VAQ).

The mean total score for the C-VAQ was found to be 31.8 (SD = 5.2, Range = 8–40) suggesting a good distribution of scores as is expected for a measure of attitudes. Cronbach’s alpha for the C-VAQ was 0.87. Internal consistency was not significantly affected if any one item was dropped. However, item level statistics suggest that the last four items should be reverse scored, which is logical given that they refer to negative attitudes about COVID-19 vaccination. Furthermore, the analysis suggested that the final item (Item 8) could be eliminated without affecting internal consistency. The polychoric correlation showed a strong to moderate correlation between items (Table 4). The scree plot analysis suggested retaining a single factor (Fig 1).

**Table 4:** Polychoric matrix between items related to attitudes toward COVID-19 vaccine.

		1	2	3	4	5	6	7	8
1	I am confident that the vaccine will protect me from COVID-19	---							
2	I feel fortunate in having access to the vaccine	.666**	---						
3	I trust the safety and effectiveness of the COVID-19 vaccine	.762**	.792**	---					
4	Benefits of the vaccine are greater than the risks	.678**	.776**	.829**	---				
5	The COVID-19 vaccine is not necessary for most people	-.470**	-.612**	-.580**	-.640**	---			
6	Natural immunity is more effective than being vaccinated	-.400**	-.473**	-.469**	-.489**	-.607**	---		
7	It is not necessary to be vaccinated if you have already had COVID-19	-.406**	-.525**	-.501**	-.544**	-.626**	-.638**	---	
8	There is not enough information about long term effects of the vaccine	-.375*	-.377*	-.484*	-.447*	.342*	.362*	.370*	---

** p < .001

*p < .05.

### Predictor model

A multiple-linear regression model was performed to predict C-VAQ scores based on the following variables: age, gender, WHO region, country income level, profession, and years of professional experience, country mortality rate, and the stringency of each country’s response to COVID-19. The regression model revealed statistically significant results for younger age (β = -.007, CI = -.012 to -.001), living in a low- or -middle income country (β = -.324, CI = -.444 to -.203), and living in an Asian country, which all predicted lower C-VAQ scores (β = -.310, CI = -.575 to -.046) suggesting more negative attitudes toward vaccination (Table 5). In contrast, having received a COVID-19 vaccine was associated with higher C-VAQ scores (β = 1.304, CI = 1.155 to 1.452) suggesting more positive attitudes toward vaccination. The number of concerns participants endorsed about COVID-19 vaccination was associated with lower C-VAQ scores (β = -.214, CI = -.233 to -.194) suggesting more negative attitudes toward vaccination. Finally, increased country mortality rate (β = -.039, CI = .004 to .075) and higher country stringency index (β = .004, CI = .001 to .007) were associated with higher C-VAQ scores suggesting more positive attitudes toward vaccination.

**Table 5:** Results of multiple-linear regression predicting C-VAQ scores (N = 1,931).

	β		CI 95%
**Personal level**			
Gender (Female)	-.017		-.093 to .059
Age	-.007*		-.012 to -.001
Years of experience (average during period)	.004		-.003 to .010
Profession (Physician)	-.041		-.126 to .043
**Behavioral level**			
Got Covid-19 vaccine (Yes)	1.304**		1.155 to 1.452
Got Covid-19 infection (Yes)	-0.029		-.146 to .088
Concerns toward vaccination	-.214**		-.233 to -.194
**Organizational level**			
Organization communicated effectively about vaccine (Yes)	.092		-.019 to .204
Organization made educational materials about vaccine (Yes)	-.007		-.128 to .115
**Environmental level**			
Income (low, lower-middle and upper-middle)	-.324**		-.444 to -.203
Region (EURO)	-.069		-.338 to .199
Region (AFRO)	-.032		-.348 to .285
Region (AMRO-North)	.117		-.159 to .393
Region (AMRO-South)	.040		-.273 to .353
Region (EMRO)	-.267		-.616 to .082
Region (SEARO)	-.204		-.495 to .088
Region (WPRO-Asia)	-.310*		-.575 to -.046
COVID-19 death rate (millions; average during period)	.039*		.004 to .075
Stringency index	.004*		.001 to .007
People vaccinated rate (per 100 000)	-.001		-.004 to .002
**Goodness of fit**			
R^2^	.42		

** p < .001

*p < .05.

## Discussion

The main findings of this study were: 1) a newly constructed measure of vaccine attitudes, the Vaccine Acceptance Quetionnaire (C-VAQ), has good internal consistency, with items loading on to a single factor, 2) having received a COVID-19 vaccine was significantly associated with higher C-VAQ scores, whereas having concerns about COVID-19 vaccination was significantly associated with lower C-VAQ scores; 3) younger age, residing in a low-and-middle income country, and living in an Asian country were all significantly associated with lower C-VAQ scores; and 4) increased COVID-19 mortality rate and higher stringency index were significantly associated with higher C-VAQ scores.

Our finding that having received a COVID-19 vaccine was significantly associated with higher C-VAQ scores provides some external validation of the measure. The measure also demonstrates some convergent validity as demonstrated by the significant negative association between C-VAQ scores and the number of concerns endorsed by participants about COVID-19 vaccination. It is also consistent with findings from experimental studies that demonstrate that changes in attitudes predict changes in health behaviours [3]. A considerably longer questionnaire assessing COVID-19 attitudes also yielded only one factor and had similar internal consistency to the C-VAQ [11]. The C-VAQ, which was translated into Chinese, English, French, Japanese, Russian, and Spanish for the present study adds to the literature a new relatively short measure that can be used by governments, organizations and researchers to efficiently measure vaccine attitudes.

Previous work has demonstrated that a range of sociodemographic factors may be associated with greater vaccine hesitancy in both health care workers and the general population [2]. It is not surprising that such sociodemographic predictors are also apparent among the attitudes of mental health clinicians toward vaccination. Nevertheless, it is important to note that there may be significant regional variation in such findings [18]. For example, within the general population, Solís Arce et al. (2021) [19] found that those living in low- and middle-income countries were more accepting of COVID-19 vaccines, particularly if guidance comes from health care workers. In the present study, we found evidence for the opposite effect such that mental health clinicians living in low- and middle-income countries as well as in Asian countries, particularly Japan, expressed more negative attitudes toward COVID-19 vaccination. This finding highlights the importance of interventions to address these beliefs in these regions. Further work must be done to understand why mental health professionals living in low- and middle-income countries, but not the general population [19], hold more negative vaccine attitudes.

It is noteworthy that higher country mortality rate and stringency index were significantly associated with higher C-VAQ scores. While environmental factors associated with vaccine attitudes may include exposure to myths about vaccines, these findings suggest that real threat from the pandemic may act to encourage positive attitudes toward vaccines. However, we cannot rule out the possibility that countries with high stringency and COVID-19 death rates had stronger programs to encourage vaccination, particularly among health professionals. A 5A model (addressing vaccine acceptability, accessibility, affordability, awareness, and activation–or nudges to use vaccine) may be useful in understanding vaccine uptake among adults [20], and in developing multi-prong interventions that address individual, organizational, and societal factors to improve such uptake [21,22].

The C-VAQ was found to possess excellent internal consistency with items loading on to a single factor. Our results suggest that the C-VAQ is a useful measure that can be used internationally to measure COVID-19 vaccine attitudes. Previous work on COVID-19 vaccine beliefs has often used a simple yes/no approach, focusing on intent to obtain vaccination [4,21]. A Likert-like measure of acceptability and hesitancy may allow for a more detailed assessment of inter-individual variations and so for more nuanced statistical analyses. A longer scale may allow assessment of different factors that contribute to vaccine attitudes, but this must be weighed against respondent burden. Future research on the C-VAQ’s sensitivity to change among international samples would be useful in the context of evaluating programs that aim to increase vaccine uptake among health care professionals.

A number of limitations deserve emphasis. First, member of the GCPN generously volunteer their time to participate in a range of research studies, and may not be systematically representative of mental health professionals. Nevertheless, the GCPN does provide access to a broad range of professionals working in diverse geographic regions and settings. Second, the design of this study does not allow determination of causality. Further work is needed to assess the precise nature of the relationships between attitudes towards COVID-19 vaccination, behavior with regards to vaccination, and social and political context. The development of the C-VAQ occurred during 2020–21 as part of an urgent call by the World Health Organization’s Global Research Roadmap [23] and funding agencies (the present study was funded by a special COVID-19 fund by the Canadian Institutes for Health Research) to address the pandemic. Furthermore, the data collection occurred at the height of the pandemic when public health measures and restrictions were in place across various countries. Variables not considered in the present report may have had a significant influence on the acceptance of vaccinations by mental health professionals at that time. While, such public measures are currently significantly less restrictive in many countries, our findings may have relevance to future pandemics. Finally, the present study examined internal consistency, factor structure, and predictive validity of the C-VAQ. Future research should validate the C-VAQ, in particular assessing convergent and divergent validity of this new measure. The availability of this measure in the published literature in advance of a future pandemic is useful. Investigating the generalizability of this brief and practical measure to other public health vaccination programs would also be beneficial. The study also has a number of notable strengths. The study includes a large sample size of GCPN members from across all five WHO regions including clinicians working in LAMICs. The multilingual nature of the study allowed for a broader participation of clinicians. Finally, the study provides the field with a brief, publicly available, free measure that can be easily administered to assess COVID-19 vaccine attitudes.

## Conclusion

Employment of the C-VAQ in the World Health Organization’s CGPN was useful in demonstrating the extent to which additional work is needed to improve mental health clinician’s attitudes towards COVID-19 vaccines around the world. Lack of vaccination in this group (7.1% of professionals reported not being vaccinated) is particularly worrisome in light of evidence that individuals with serious mental illness may be heightened risk of infection and increased mortality [5]. Furthermore, protection of an already fragile mental health workforce is a priority particulary with evidence of growing prevalence of mental health conditions during the pandemic (e.g., [24]). While increased intervention efforts may be particularly pertinent for a number of sociodemographic groupings, relatively high levels of vaccine hesitancy in clinicians and communities around the world also suggest the need for broad, multi-pronged interventions that address negative attitudes among large numbers of individuals in future pandemics.
